# The Role of Biomaterials in Peripheral Nerve and Spinal Cord Injury: A Review

**DOI:** 10.3390/ijms23031244

**Published:** 2022-01-23

**Authors:** Ben Kaplan, Shulamit Levenberg

**Affiliations:** 1 Faculty of Biomedical Engineering, Technion-Israel Institute of Technology, Haifa 3200003, Israel; kap@campus.technion.ac.il; 2 Bruce Rapaport Faculty of Medicine, Technion-Israel Institute of Technology, Haifa 3525433, Israel

**Keywords:** spinal cord injury, peripheral nerve injury, biomaterials

## Abstract

Peripheral nerve and spinal cord injuries are potentially devastating traumatic conditions with major consequences for patients’ lives. Severe cases of these conditions are currently incurable. In both the peripheral nerves and the spinal cord, disruption and degeneration of axons is the main cause of neurological deficits. Biomaterials offer experimental solutions to improve these conditions. They can be engineered as scaffolds that mimic the nerve tissue extracellular matrix and, upon implantation, encourage axonal regeneration. Furthermore, biomaterial scaffolds can be designed to deliver therapeutic agents to the lesion site. This article presents the principles and recent advances in the use of biomaterials for axonal regeneration and nervous system repair.

## 1. Introduction

Biomaterials have been extensively studied and tested as tools and treatments in regenerative medicine and tissue engineering. Due to their biological activity, they can facilitate tissue repair and serve as carriers of cellular and pharmacological treatments [1,2]. Tissue regeneration has been demonstrated in various types of injuries and diseases following biomaterial-based treatment, including in the nervous system where injuries have potentially devastating consequences [3,4].

This article reviews some of the principles and recent advances in the research on biomaterials for nervous system regeneration and repair. Specifically, we will address attempts to utilize biomaterials for peripheral nerve injury (PNI) and spinal cord injury (SCI). Despite the different biology of these two conditions, in both cases, the disruption of axonal fascicles in a process of Wallerian degeneration is a primary cause of neurological deficits [5,6]. In Wallerian degeneration, axons degrade and eventually vanish at any point distal to the lesion [7]. However, cell bodies, along with their proximal segment of axon, may survive [8]. Hence, PNI and SCI experimental treatments have sought to induce the regeneration and elongation of surviving proximal segment axons. Over the past few years, advances have been made in injury models of PNI and SCI. Many of the developed biomaterial interventions and treatments are suitable for both types of injuries and indeed, some have been tested for both.

## 2. Peripheral Nerve Injury

**Figure 1:**
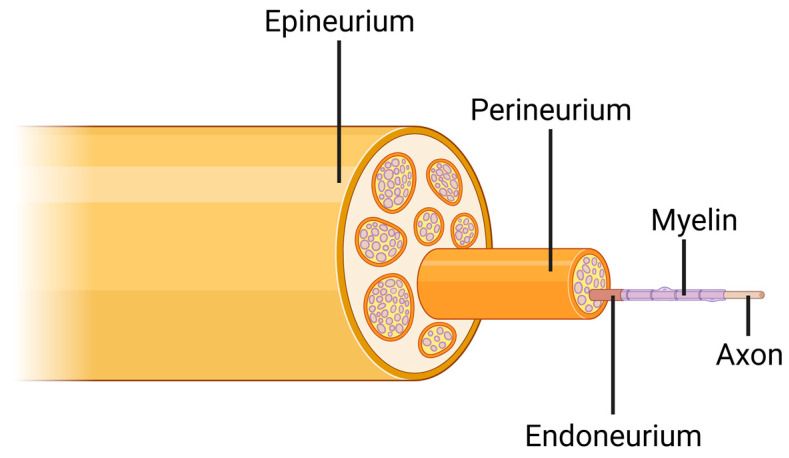
Inner structures of a peripheral nerve. The entire nerve is surrounded by the epineurium collagenous membrane. Each fascicle of axons is separated from surrounding tissue by its own perineurium sheath. Each single axon, along with its myelin sheath, is coated by a delicate endoneurium membrane. Created with BioRender.com.

The peripheral nervous system (PNS) connects the brain and spinal cord to target tissues and organs throughout the human body [9]. Each nerve is comprised of several fascicles, measuring up to several hundred microns in diameter [10]. Every fascicle contains numerous axons and Schwann cells, which extend myelin sheaths around many different types of axons [11]. Peripheral nerves also contain three layers of collagenous connective tissue [12] (Figure 1). The endoneurium is a delicate intrafascicular connective tissue in close proximity to the myelin sheets of the axons. The perineurium is a protective membrane of collagenous tissue that ensheathes each nerve fascicle. Both the endoneurium and perineurium provide elasticity to the nerves. The epineurium is a sheath of connective tissue that surrounds the entire nerve structure. Together, these three membranes of collagen define the micro-architecture and topography of the nerve. Additionally, they serve as pathways for blood supply, as blood vessels run along the epineurium and perineurium, and extend capillaries into the endoneurium [12].

**Figure 2:**
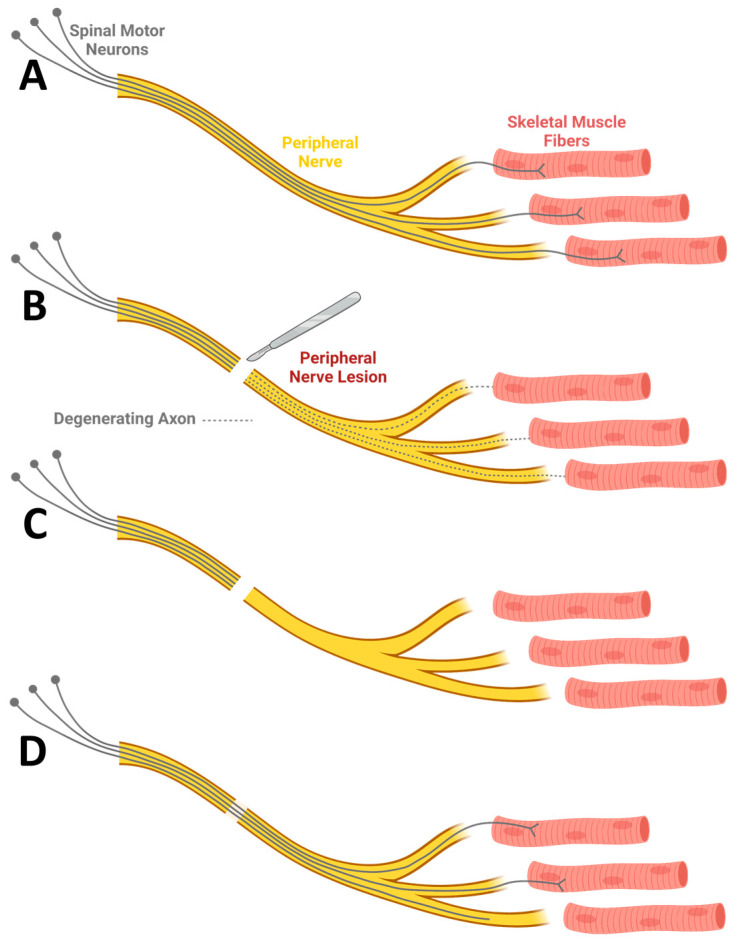
Axonal degeneration and regeneration in peripheral nerve injuries. (**A**) Cell bodies of spinal motor neurons reside in the spinal cord and extend axons throughout intact peripheral nerves to innervate skeletal muscle fibers. (**B**) Following transection of axons, axonal segments located distal to the lesion site will undergo Wallerian degeneration. (**C**) Following axonal degeneration, spinal motor neurons and skeletal muscle fibers may survive; however, the connectivity between these groups of cells is lost. (**D**) Depending on the severity of injury, regeneration and partial or complete reinnervation of skeletal muscle fibers may take place. Created with BioRender.com.

Trauma accounts for 87% of PNI cases [12]. PNI is most common in the upper extremities, where over 80% of all nerve injuries occur [12]. The clinical management of PNI is complex and depends on the severity of injury. Neuropraxia, in which only myelin membranes are affected, and axons remain intact, is the mildest type of nerve injury. In this case, spontaneous remyelination and recovery are expected [11]. Axonotmesis is a more serious type of injury in which axons are by definition severed and undergo Wallerian degeneration [11] (Figure 2A–C). In mild axonotmesis, the endoneurium and perineurium membranes remain intact. In severe axonotmesis, these inner membranes are disrupted and only the outer epineurium membrane remains. Neurotmesis, which involves a complete transection of the nerve structure with obliteration of all three collagenous membranes along with axonal transection and degeneration, is the most severe type of nerve injury [11].

Following injury, the axons of the PNS can regenerate and may facilitate functional recovery (Figure 2D). The regenerative capacity of peripheral axons is obtained by cell-intrinsic molecular mechanisms. Injury to the peripheral nerves triggers axon-to-cell body-retrograde signals, which activate the expression of several regeneration-associated genes. For example, Atf3, Jun, Hsp27, Sprr1a, Gap43, and JAK-STAT pathway-related genes have been found to be associated with axonal regeneration and their expression was induced following injury [13]. Within hours post-injury, a growth cone assembles at the leading edge of the axon and guides axonal elongation [14]. The growth cone is a sensory motile structure, possessing several types of cell membrane receptors capable of binding to cellular and extracellular molecules. In parallel, cell-extrinsic mechanisms operate to support regeneration. Severed axons trigger an inflammatory response, which activates Schwann cells. Schwann cells dedifferentiate into progenitor-like cells, proliferate, and, along with macrophages, phagocytose debris and clear the way for axons to regenerate [11,15]. They then form tubes of progenitor-like cells onto which regenerating axons can attach and grow [11].

Despite the intrinsic and extrinsic mechanisms that allow peripheral axonal regeneration, not all PNIs heal spontaneously [11]. In mild axonotmesis, the spared endoneurium and perineurium are sensed by Schwann progenitor-like cells and regenerating axons, which follow the architecture of the connecting tissue. The connecting tissue membranes essentially guide axons across the lesion site and to their pre-injury targets. This type of injury can thus heal spontaneously. In severe axonotmesis and neurotmesis, the absence of structural collagenous membranes interferes with the healing process [11]. In the absence of structural cues to guide axons, the intrinsic axonal capacity to regenerate tends to take place as disorganized growth. In addition to not reaching pre-injury targets, axons that grow in a disorganized fashion will form painful neuromas [11].

To avoid the complications of disorganized axonal regeneration and neuroma formation, nerve autograft implantation is often required in severe cases of PNI [16]. Autologous nerve grafting essentially requires sacrificing a healthy nerve to treat a more vital, compromised nerve [16]. When nerve autografts are harvested, axons within them degenerate. However, their structural collagenous membranes remain intact, and, upon implantation, they facilitate the guidance of axonal regeneration throughout the lesion site. Autografts, however, have several limitations. In large-gap injuries (>5 cm), their efficiency is restricted, their availability is limited, and the harvesting procedure may generate sensory deficits [17,18]. Additional donor site morbidities include scarring and possible neuroma formation [17].

## 3. Spinal Cord Injury

**Figure 3:**
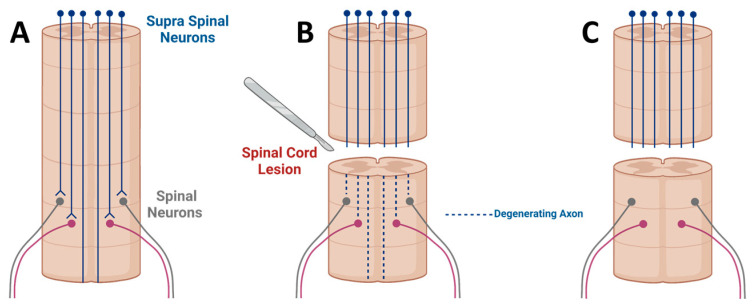
Axonal degeneration following spinal cord injury. (**A**) In the intact spinal cord, supra spinal neurons, located in the brain, extend long tract axons into the spinal cord. These axons synapse onto spinal neurons, which extend axons into the peripheral nervous system. (**B**) When the spinal cord sustains an injury, axons are severed and undergo Wallerian degeneration distal to the lesion site. (**C**) Following degeneration, supra spinal neurons and spinal neurons often survive; however, their connections are lost. In contrast to peripheral axons, injured axons in the spinal cord do not exhibit meaningful regeneration across the lesion site. Created with BioRender.com.

SCI is a traumatic syndrome with devastating effects on patients’ lives. It is estimated that over 130,000 people are affected by SCI each year, worldwide [19]. Approximately 45% of all injuries are complete [20], leaving but a slight chance for functional recovery. Neurological deficits in SCI are the result of both primary injury to the tissue and a cascade of biological events that follows, referred to as ‘secondary injury’ [21]. Secondary injury events include inflammation, vascular changes, free radical formation, disruption of ionic balance, and glutamate toxicity, all of which contribute to additional neuronal damage [21]. The process of injury leads to neuronal cell death at the site of injury but, more importantly, to the disruption of spinal cord white matter [5]. Spinal cord white matter is comprised of several tracts. Each tract is a bundle of axons that delivers signals to or from the brain [21]. When the spinal cord sustains an injury, axons of the tracts are often disrupted and Wallerian degeneration occurs [22] (Figure 3A,B). Despite the possible survival of cell bodies and proximal axonal segments, neuronal signals cannot be transmitted beyond the lesion [8] (Figure 3C).

To date, patients who suffer from SCI receive mostly palliative treatment [21]. The interventions currently used to facilitate recovery are surgical decompression of the injury site and methylprednisolone administration [23,24]. Both treatments are considered limited in their effect on functional recovery [21]. Efficient interventions with the potential to induce significant regeneration and restoration of function are still lacking in the clinical setting. Unlike neurons of the PNS, which are capable of regrowing their axons from the site of injury, axons of the adult spinal cord have very limited growth capacity [25]. When functional recovery is observed following a partial SCI, it is generally attributed to rehabilitation and plasticity rather than to regeneration through the lesion site [26]. The discrepancy between the regeneration capacity of the PNS and the almost entirely absent regeneration capacity of the spinal cord raises a fundamental question. What, then, is the source of growth inhibition in the adult spinal cord? This crucial question has motivated extensive research over the past several decades. Studies have identified a number of causes for regenerative failure, which we discuss below.

Spinal cord injuries often result in the formation of cystic cavities filled with cell debris and fluid [27]. The lack of permissive substrates within these cavities prevents the extension of axons into the lesion, and the axons fail to attach to non-permissive substrates [28]. For proper attachment and elongation, axons require growth-permissive substrates, normally in the form of an extracellular matrix (ECM), which contains adhesion mediating molecules such as fibronectin, laminin and collagen [29]. In addition to the absence of adhesion molecules at the lesion site, the local production of growth factors is also scarce. In the PNS, Schwann cells increase the secretion of essential neurotrophic factors such as nerve growth factor (NGF) and brain-derived neurotrophic factor (BDNF) in response to injury and support regeneration [30]. However, the production of these molecules and other neurotrophic factors in SCI lesions is limited [31,32].

In the injured spinal cord, the absence of molecules required for regeneration is compounded by the presence of inhibitory molecules. These inhibitory molecules are capable of binding to receptors that are expressed on cell membranes of axons and affect intracellular signaling cascades [21]. The first group of inhibitory molecules are myelin- associated inhibitory proteins, including Nogo, myelin-associated glycoprotein, oligodendrocyte myelin glycoprotein, semaphorin, and ephrin [32,33,34]. Attempts to block the effects of these molecules with antibodies or genetic manipulations have resulted in some degree of axonal regeneration [35,36,37]. The second group of inhibitory molecules are the chondroitin sulfate proteoglycans (CSPGs) associated with the spinal ECM, which are produced mostly by astrocytes, oligodendrocytes precursors, and meningeal cells [21]. The CSPGs include Agrecan, Brevican, Neurocan, NG2, Phosphacan, and Versican [21]. These molecules are present in the intact and healthy spinal cord. However, following an injury, their local expression is increased, especially within the glial scar formed following an injury [38]. Degrading CSPGs by enzyme delivery to the lesion site has helped to promote regeneration and some degree of functional recovery [39].

Physical barriers also seem to play a role in growth inhibition. The inflammatory process that follows the injury is restricted by a scar formation, termed the ‘glial scar’ or ‘astrocytic scar’ [32]. The scar is composed mainly of reactive astrocytes, immune cells and CSPG molecules [40]. This scar acts as a physical barrier for regenerating axons approaching the lesion site and was once considered strictly inhibitory [21,32]. The attenuation of scar formation has been reported to be beneficial for achieving axonal regeneration [32]. However, more recent studies have shown a beneficial role for the scar and demonstrated that there is additional damage when it is eliminated [41]. Despite its inhibitory nature for axonal regeneration, the glial scar is considered necessary for the prevention of additional damage to the neural tissue from inflammation and toxicity that are present after SCI [40]. The scar achieves this by forming a limitans border of astrocytes between the neural tissue and the lesion core and restricting the invasion of inflammation deeper into neural tissue [32]. Moreover, several studies have shown that axons are capable of growing throughout the glial scar despite its inhibitory nature [41,42].

In addition to the above-mentioned cell-extrinsic inhibitors of regeneration, intrinsic inhibition plays a significant role in spinal cord regeneration failure. Substantial differences in gene expression between PNS and CNS neurons have been reported [43]. Particularly, regeneration-associated genes have been shown to be induced following PNI but not after SCI. Several studies have demonstrated that reactivation or downregulation of specific genes such as PTEN, mTOR, and SOCS3 contributed to significant growth, including impressive regeneration of the highly refractory corticospinal tract axons [44,45]. Additionally, grafted neural stem cells (NSCs) which differentiated into early, embryonic-like neurons were able to grow to extremely long distances in a complete SCI despite the presence of scars and inhibitory molecules [46]. Recently, it was reported that the growth of such early neurons is enhanced by adult myelin despite (and possibly due to) the presence of proteins considered inhibitory [47,48]. This observation may suggest that the effect of molecules considered inhibitory for growth is dependent on the expression of certain neuronal receptors that may be upregulated only in adult neurons. Therefore, intrinsic inhibition may also control the interaction of axons with the inhibitory extrinsic environment.

## 4. Axonal Interaction with Biomaterials

Spinal and peripheral axonal regeneration necessitates the binding of cell membrane receptors to components of the ECM. Integrins are heterodimeric cell membrane receptors that anchor cells to ECM proteins [49]. Of the 24 different integrins that have been identified thus far in humans, many are expressed at high levels in the developing nervous system. However, most integrins are downregulated in adult neurons. Integrins are also present at the growth cones of regenerating axons, where their binding to permissive substates facilitates axonal regeneration [50]. Several important ECM molecules present in the nervous system can bind to integrin receptors. For example, collagens are ECM proteins that provide structural support and tensile strength to tissues but also affect cell migration. They are widely present in the CNS and PNS, and during development they bind to integrin-expressing growth cones [49]. Laminins, another type of permissive ECM molecule, are large glycoproteins that form a major part of the nervous system’s ECM [49]. Fibronectin is also widely expressed in the nervous system. The arginine-glycine-aspartic acid (RGD) domain in fibronectin can bind to several different types of integrins and promote axonal outgrowth [51].

Extracellular proteins play a key role in the regeneration of axons. Both PNI and SCI injuries are characterized by inadequate physical and molecular substrates to which axons can attach. In PNI, destruction of the structural collagenous membranes generates misguided and inefficient regeneration, whereas in SCI, axons are unlikely to demonstrate any growth to the cystic lesion site [11,52]. Biomaterials can offer a solution to overcome these limitations as they can be designed to mimic ECM architecture and serve as substrates onto which axons can attach. Some biomaterials are made from the ECM proteins themselves. Other biomaterials can be processed to contain ECM proteins to facilitate cell attachment.

Biomaterials are generally divided into two categories: natural and synthetic. Natural biomaterials can be assembled from ECM proteins. For example, collagen hydrogels and laminin hydrogels have been utilized for various applications of tissue engineering, including PNI and SCI [53,54,55,56]. ECM-based biomaterials tend to integrate well into injured tissue and are permissive for axonal outgrowth. However, ECM hydrogels usually degrade rapidly in vivo, and have low mechanical strength [57]. Natural biomaterials also include non-ECM materials. For example, alginate can be produced from algae, and chitosan can be produced from the outer skeleton of shellfish [58]. Their degradation period can be tuned to match specific needs. However, they are assembled from non-permissive molecules and cannot specifically interact with mammalian cells [59,60].

Synthetic biomaterials include polylactic acid (PLA), poly lactic-co-glycolic acid (PLGA), polycaprolactone (PCL), polyethylene glycol (PEG), and many others. Their properties can generally be tuned more easily than natural biomaterials. For example, porosity, rigidity, and degradation rate can be altered to match different types of tissues [61,62]. However, synthetic biomaterials may require ECM coating or other surface modification as they do not contain integrin-binding molecules [57].

**Figure 4:**
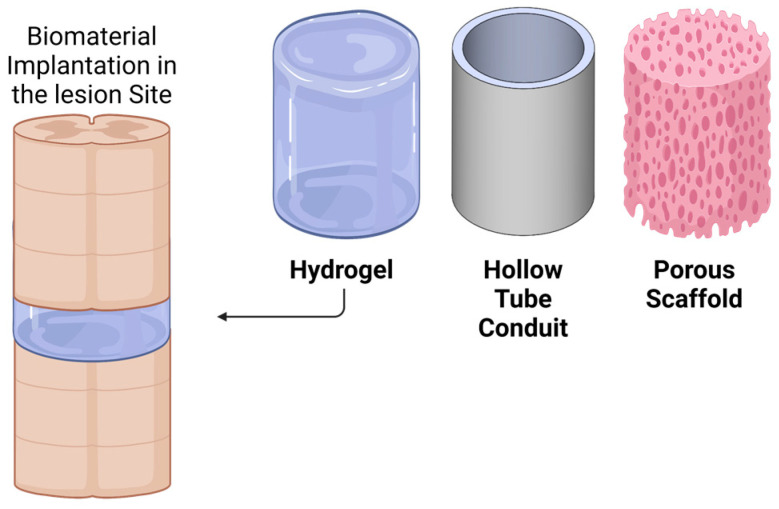
Types of biomaterial implants for nerve injuries. Hydrogels are injectable biomaterials that can solidify upon implantation and conform to irregular lesion sites. Hollow tube conduits are often used in peripheral nerve injuries; however, they do not contain inner structures to mimic the collagenous membrane of nerve tissues. Porous scaffolds provide better mechanical stability and longer degradation periods compared with hydrogels. Their inner architecture can be designed to mimic the nerve tissue ECM. Created with BioRender.com.

Several different biomaterial implants can be delivered to a PNI or SCI lesion site (Figure 4). Hydrogel biomaterials can be designed to solidify in situ after injection as liquids to the lesion site. The solidification can be induced by mixing two reacting molecules or by change of temperature, as some hydrogels can be designed to be thermoresponsive and solidify at body temperature [46,63,64]. Injectable hydrogels are particularly helpful in experimental treatments for SCI where lesion sites are small, irregular and confounded by the spinal cord meninges. Injectable hydrogels conform to the irregular shape of the lesion and provide ideal contact between the severed stumps of the spinal cord and the biomaterial. Many different hydrogels have been used in models of SCI. For example, a series of studies by the Tuszynski lab used injectable fibrin hydrogel as part of a neural stem cell therapy for complete and partial SCI [46,65,66]. Combining fibrin matrices and growth factors dramatically increased the number of surviving stem cells in the spinal cord [67]. Recently, Li et al. developed a thiol-modified hyaluronic acid hydrogel conjugated to fragments of synthetic PCL in a contusion SCI model [68]. The incorporation of PCL into the hydrogel provided mechanical support for the construct. It also contributed to the formation of new tissue at the lesion site, including blood vessels and regenerating axons. It is possible that the improved properties observed in this specific biomaterial were due to slower degradation as a result of PCL incorporation in the hydrogel. However, this subject requires further investigation.

Hollow tube conduits are another form of transplantable biomaterial for axonal regeneration. This biomaterial tube, which serves as a bridge between severed stumps of nerve tissue, is used more often in PNI than in SCI [69,70]. The tube can be fabricated from various biomaterials, such as collagen and PLGA [69,71]. The conduit isolates the lesion site from the surrounding environment and prevents misguided regeneration outside the route of the peripheral nerve. However, since hollow tube conduits do not mimic the inner perineurium and endoneurium, there can still be some disorganized growth inside the tube. This may explain why hollow tube conduits are less effective than nerve autografts in large-gap peripheral injuries [72]. The regeneration depends heavily on the proliferation of Schwann cells within the tube and their guidance of axonal growth, which may not be as efficient in the absence of the endoneurium membrane. For small-gap peripheral injuries, however, hollow tube conduits do seem to be effective. Furthermore, hollow tubes can be pre-loaded with hydrogels to improve their biological function [73].

Porous scaffolds provide a more advanced form of biomaterial compared with hollow tube conduits and have been tested both in PNI and SCI models. They can be made from various polymers, both natural and synthetic. These scaffolds tend to provide better mechanical strength than hydrogels, and, when fabricated from synthetic materials, their degradation period can be tuned to last for longer periods [61]. As with hollow tubes, porous scaffolds can isolate the lesion site from surrounding tissue. However, the inner microarchitecture of the pores can be designed to mimic the ECM of nerve tissues and encourage axons to grow in their pre-injury directionality in order to avoid disorganized growth [74]. Axons which penetrate porous scaffolds grow within their pores while they use the inner scaffold walls as attachment surfaces. As in the case of hollow tubes, many examples exist for preloading with hydrogel to improve biological activity [62,74]. Seeding stem cells in porous scaffolds prior to implantation has also been shown to positively affect vascularization sensation and locomotion in models of complete SCI [75,76]. Despite their efficiency in regenerating axons, porous scaffolds have several limitations. First, their geometry is determined prior to implantation, and, unlike hydrogels, they cannot conform to irregular lesion sites. This may generate small gaps in the scaffold-tissue interface where axons do not meet a permissive substrate. Second, the presence of an inner wall microarchitecture reduces the open volume into which axons can regenerate. For example, some microarchitectures that were found to be very useful for axonal guidance are difficult to produce with an open volume of more than 45%, which limits the number of axons that can penetrate the lesion site [61].

## 5. Surface Modification

The absence of permissive molecules on the surfaces of synthetic biomaterials has led to the use of surface modification techniques to enhance their bioactivity for both PNI and SCI implants. In these processes, ECM proteins and many other biomolecules are used to modify surfaces of synthetic biomaterials. Surface modification can affect the performance of biomaterials in various ways, including reduction in immunological response and increase in cell attachment [61,77].

Several techniques for surface modification have been developed for such purposes. Physical adsorption is one of the simplest techniques to modify biomaterial surfaces. Biomaterial substrates are immersed in solutions containing ECM proteins or other biomolecules. The biomolecules then bind to the biomaterial substrates in non-covalent binding. Such binding can take place via electrostatic force, hydrophobic bonds, van der Waals interactions or hydrogen bonds [78]. As an example, Shahriari et al. developed a PCL scaffold for complete SCI, coated with fibronectin using physical adsorption [61]. The adsorption enhanced the attachment of fibroblasts and Schwann cells to the scaffold walls in vitro, potentially increasing the suitability of its surface for axonal attachment and regeneration.

Chemical modification techniques are another approach to bind permissive proteins to biomaterials substrates. The main difference between chemical and physical modification is the generation of covalent bonds between the permissive proteins and scaffold surface in the former. As a result, protein binding to the biomaterial endures for longer periods of time [78]. Consequently, the biological activity may be enhanced. For example, Manchineella et al. showed that chemical modification resulted in an increased proliferation in vitro of stem cells on the biomaterial surface compared with physical modification [79]. In another study, a series of chemical reactions were used to attach laminin to the surface of slow-degrading silica nanofibers. The modified scaffold was compared to silica nanofibers that were physically adsorbed with laminin in in vitro experiments. The chemically bound nanofibers were able to retain laminin significantly longer and enhanced neurite outgrowth. However, in vivo studies have not been carried out [80].

While many studies show a clear benefit in vitro for modifying synthetic biomaterial surfaces with ECM proteins in cell attachment and growth, findings to support these modifications for in vivo implantation in peripheral nerves or in the spinal cord are not as clear. Several studies that examined surface modifications for nerve injuries were restricted to in vitro cellular assays, or performed in vivo implantation without a non-functionalized biomaterial control group [61,81,82]. In contrast, Novikova et al. implanted a hydroxybutyrate scaffold loaded with Schwann cells in an injured spinal cord [83]. In this study, physical adsorption with fibronectin prior to implantation did not improve in vivo axonal regeneration.

The use of proteins for surface modifications has several disadvantages. They may elicit an undesired immune response and undergo proteolytic degradation [84]. Consequently, their ability to endure in vivo implantation over prolonged periods of regeneration may be limited [84]. In addition, the interaction of ECM proteins with biomaterial surfaces can influence the conformation of proteins and limit the availability of cell-binding motifs [84]. These limitations can be overcome to some degree by synthesizing cell-recognition motifs as small cell adhesive peptides capable of binding to integrin receptors. Cell adhesive peptides tend to be more stable than full-length proteins. Additionally, ECM proteins contain several different cell-recognition motifs while cell adhesive peptides contain only one. Therefore, they can facilitate cell receptor binding more selectively [84]. The RGD sequence peptide is very effective and the most widely used peptide for synthetic surface modification. It was first identified in fibronectin as an integrin-binding domain and later in vitronectin, laminin, collagen, and various other proteins [84]. Its discovery led to its inclusion in many PNI and SCI studies. As an example, a blend of RGD peptide-conjugated polyurea and PCL was used to fabricate a peripheral nerve scaffold. The scaffold was tested in vivo in a large 10 mm gap sciatic nerve injury and was assessed in electrophysiology and immunostaining. The RGD- conjugated scaffold was shown to outperform a PCL-only scaffold and even showed similar or improved characteristics compared with an autograft [85].

Following the discovery of RGD, many different ECM-derived peptides were synthesized and explored for various purposes of tissue engineering. The laminin-derived peptides IKVAV and YIGSR have both been shown to support axonal outgrowth [57]. For example, Zhu et al. showed that combined non-covalent binding of RGD and YIGSR generated a synergistic effect on Schwann cell proliferation and neurite outgrowth in vitro and increased axonal regeneration in a model of PNI [86]. More recently, Sever-Bahcekapili et al. used self-assembling peptide molecules, designed to form nanofibers, which display heparan sulfate and laminin mimetic epitopes to assemble a scaffold for spinal cord repair. The scaffolds were reported to facilitate functional recovery after implantation to a spinal cord lesion [87].

## 6. Biodegradation

The degradation of implanted biomaterials is essentially the cleavage of polymer chains to oligomers and monomers. This can take place via several mechanisms, including water-induced hydrolysis and enzymatic degradation [88]. Degradability is often considered a necessary requirement for biomaterial scaffolds to avoid the permanent presence of a foreign body. Ideally, biomaterial degradation products should be nontoxic and able to exit the body via metabolic pathways [89]. As the duration of degradation varies considerably between different biomaterials, it is important to use an appropriate biomaterial to match the time frame of recovery. An implanted scaffold should remain stable, without the collapse of its inner architecture, for a time frame that matches the process of healing [90].

Regarding experimental models of SCI, scaffolds should be designed to remain stable at least 4 weeks in vivo in order to provide adequate support for axons regenerating across the lesion site [91]. Peripheral axons regenerate at a rate of 1 mm a day, and this rate, along with the size of injury, which may vary between different injury models, should be considered when choosing a biomaterial for PNI [11,72]. Several studies have demonstrated the importance of an adequate degradation rate. When using alginate scaffolds in a complete SCI, rapid degradation was observed, and, at a time frame of two weeks, hardly any regeneration was observed due to collapse of the scaffold walls [92]. However, use of a similar scaffold structure with agarose or PEG-Gelatin methacrylate (GelMA) resulted in a stable architecture which facilitated increased axonal regeneration at four weeks post-injury [91]. The PEG-GelMA scaffold was also evaluated at six months post-implantation, where some evidence for degradation was observed in histology.

Degradable polyesters are some of the most widely used biomaterials in tissue engineering [93]. One of their clear advantages is the convenient tuning of degradation rate, which can range from days to years [62]. The molecular weight, crystallinity and type of polyester can be chosen to tailor the degradation period for a specific need. Several different polyesters can also be mixed to optimize the degradation rate. For example, rapidly degrading low-molecular PLGA was mixed with slowly degrading poly-L-lactic acid (PLLA) to achieve a scaffold with a degradation period of several months [94]. Polyesters have also been widely used for spinal cord and peripheral nerve injuries. For example, a PCL multi-channel scaffold was used both in SCI and PNI, where it underwent excellent integration into the host tissue, supported axonal regeneration and remained stable throughout the entire study period [61,95]. PLGA multi-channel scaffolds were also tested in a partial SCI model, where they were shown to support the regeneration of axons into the lesion site and beyond it, back to intact tissue. This regeneration was also associated with improved forelimb function [96].

## 7. Foreign Body Response

Every implantation of a biomaterial scaffold for tissue regeneration will trigger an inflammatory response [77]. This response can vary considerably, but in some cases it can result in implant failure. The inflammatory response is characterized by protein adsorption, especially C3 and C5 from the complement family [77]. The adsorbed proteins then serve as chemoattraction gradients for immune cells, including macrophages. When this process escalates and macrophages fuse into a foreign body giant cell, the inflammation is classified as a foreign body response [77]. This immunological reaction generates a fibrous capsule around the implant and isolates it from the host tissue [97].

**Figure 5:**
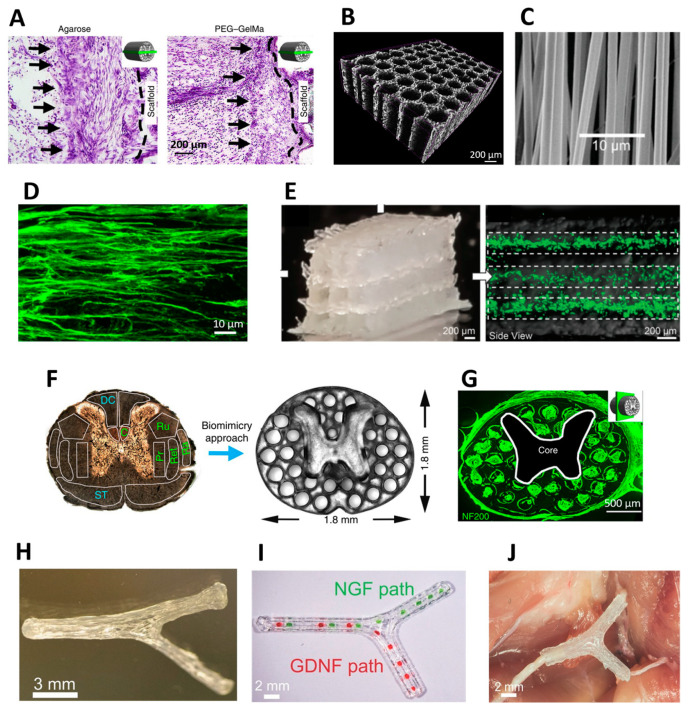
Examples of biomaterial scaffolds for spinal cord and peripheral nerve injuries. (**A**) differences in size of a foreign body encapsulation around an agarose scaffold (left) and a polyethylene glycol (PEG)- Gelatin methacrylate (GelMA) scaffold implanted in a complete spinal cord injury model in rats [91]. Reproduced with permission. Copyright 2019, Springer Nature. (**B**) Example of polyester microchannel scaffold. The structure was scanned and imaged using micro-CT [62]. Reproduced with permission. Copyright 2020, Elsevier. (**C**) Example of a fibrous scaffold for spinal cord repair composed from GelMA and fabricated using electrospinning [99]. Reproduced with permission. Copyright 2019, John Wiley and Sons. (**D**) neural stem cell derived axons which grow inside a single microchannel adopt a linear growth pattern according to the scaffold topography [91]. Reproduced with permission. Copyright 2019, Springer Nature. (**E**) Bioprinted alginate scaffold containing accurately deposited neural progenitor cells in each microchannel [100]. Reproduced with permission. Copyright 2018, John Wiley and Sons. (**F**) 3D printed PEG-GelMA scaffold for spinal cord repair inspired by the native architecture of the spinal cord. (**G**) Upon implantation NF200 positive axons penetrate the 3D printed microchannels [91]. Reproduced with permission. Copyright 2019, Springer Nature. (**H**) Anatomically inspired 3D printed scaffold to reconstruct the sciatic nerve bifurcation. (**I**) In each branch, a different growth factor gradient is located to match the specific type of axons for each pathway. (**J**) In Vivo implantation of the anatomical scaffold [101]. Reproduced with permission. Copyright 2015, John Wiley and Sons.

Regarding SCI, Gros et al. used multi-channel agarose scaffolds that induced impressive, aligned regeneration in partial SCI lesions. However, over time, a foreign body response was observed in the scaffold-spinal cord interface which seems to have blocked regenerating axons from exiting the scaffold into healthy cord tissue. Axons were essentially trapped in the lesion site and were not able to reconnect with spinal neurons [98]. These results were reproduced in a later study by the authors in which the agarose scaffold was compared to a PEG-GelMA scaffold with similar topography. The PEG-GelMA biomaterial was shown to be superior and evoked a minimal inflammatory response. A layer of reactive cells was observed around it, but it was significantly thinner than that formed around the agarose scaffold [91] (Figure 5A). Additionally, astrocytic processes were able to penetrate the PEG-GelMA scaffold, whereas in the agarose scaffold they remained at the scaffold-host interface and were restricted by the foreign body response. The PEG-GelMA scaffold was later implanted with NSCs and regeneration across the entire lesion site was observed, with penetration of regenerating axons into the host spinal cord. This study demonstrated how an inadequate biomaterial can restrict axonal regeneration in a foreign body response mechanism.

While different classes of biomaterials provoke different immunological responses, there are several ways to reduce the inflammation associated with a specific biomaterial. For example, in a mechanism that is not entirely clear, surface modification with ECM proteins may attenuate the inflammatory response [77]. Swartzlander et al. showed that PEG hydrogels functionalization with RGD molecules decreased fibrous encapsulation compared with non-functionalized controls [102]. Another strategy to reduce the inflammatory response is to process the biomaterial with higher porosity [77]. Porous biomaterials demonstrate reduced encapsulation. Several recent studies have incorporated additional, smaller-scale pores between the scaffold’s guidance channels to generate nerve-like mechanical properties [61,62]. This strategy may also be beneficial for reducing the foreign body response.

## 8. Topography-Mediated Axonal Guidance

The elongation of axons into appropriate regions in the developing spinal cord is mostly mediated by chemical signals [103]. Secreted axonal guiding molecules form gradients of chemoattraction or chemorepulsion. These gradients can guide growing axons to their targets by binding to their matching receptors expressed on the membranes of axonal growth cones found at the distal ends of regenerating axons [103,104]. The Netrin-1 protein is considered one of the most important guidance molecules in the nervous system. The chemoattraction of Netrin-1 is mediated by the deleted in colorectal cancer (DCC) protein, a Netrin-1 receptor. This receptor is responsible for guidance of axonal growth in the embryo toward a gradient of Netrin 1 peptide [103]. During the embryonic period, DCC is expressed in the growth cones of neurons, including in supra-spinal neurons, and soluble Netrin 1 serves as a long-range cue for growing axons by creating gradients of chemoattraction [105]. Many other chemoattraction or chemorepulsion proteins have been identified, including slits, semaphorins, and ephrins. However, in the adult nervous system following an injury, the majority of chemical guidance cues are absent, and axons may grow in a disoriented fashion [106].

The disoriented regeneration of axons generates a number of obstacles:

(1) Most importantly, in the absence of guidance, the number of axons that will reach their pre-injury targets will be very limited [98]. For example, with lack of guidance following peripheral nerve injuries, axons will regenerate; however, only a small fraction of them, if any, will reach the denervated muscle fibers.
(2) In case axons do regenerate a long distance into their denervated targets, disoriented growth may lead them to inappropriate targets [74]. For example, motor axons may regenerate into sensory regions.
(3) Disoriented growth from the end of a severed nerve will generate bulb-like structures known as neuromas, which consist of many disoriented axons and, via mechanisms not clearly understood, cause severe neuropathic pain [106].

As with other types of cells, growing axons of neurons can sense topographic cues in their environment and grow in the direction of these cues [107]. This key principal is being applied in tissue engineering in order to organize cells to grow in the appropriate directionality [107]. To utilize this principal in SCI and PNI, scaffolds can be fabricated in various ways to mimic the microanatomy of nerve tissue and guide axons to aligned regeneration [108]. Polymeric scaffolds that support linear and aligned axonal growth have been shown to help regenerating axons grow in accordance with their appropriate functional and anatomical organization and increase the quantity of regenerating axons in SCI and PNI models [74,109]. Without linear guidance, it is very difficult for axons to regenerate across large lesions even when permissive substrates and neurotrophic support are provided [98]. The addition of topographic guidance can promote growth of up to nearly 85% of regenerating axons across the entire lesion [98].

Several different aligned topographies have been designed and tested for mimicking the microarchitecture of nerve tissue. Common topographies used in in vivo studies are microchannels and microfibers (Figure 5B,C). Microchannel scaffolds have been extensively used and tested for both SCI and PNI. Various fabrication techniques have been employed, including templating and freeze-drying [74,110]. Microchannel scaffolds include multiple channels with a typical diameter of 100–300 µm, similar in size to axonal fascicules in the spinal cord or in peripheral nerves [74]. Multiple axons penetrate each channel with remarkable linear directionality (Figure 5D). However, microchannel scaffolds are significantly limited in their open volume for axonal penetration. The scaffold walls which compartmentalize each microchannel are non-penetrable for axons. The walls typically comprise over 40% of the scaffold volume and therefore drastically limit the quantity of axons that can regenerate through the scaffold [61].

Aligned fibrous scaffolds have also been widely used in experimental SCI and PNI. These scaffolds are comprised of micron-scale fibers that can be produced from a wide variety of biomaterials, both synthetic and natural [111,112,113]. The main fabrication method for fibrous scaffolds is electrospinning. Axons attach to fibers and grow in response to their directionality. As an example, Chen et al. recently used electrospinning to fabricate a scaffold from GelMA fibers which was tested in a partial SCI model [99]. The generated scaffold mimicked the spinal cord rigidity and guided cell growth in linear trajectories due to the aligned topography. The high elasticity of the scaffold was also an advantage and allowed the implant to withstand implantation to the injury site without being permanently deformed. Recently, Dong et al. was able to elucidate the cascade of biological events of peripheral nerve regeneration in response to a fibrous scaffold implantation [114]. The aligned fibers recruited macrophages and subsequently facilitated polarization toward a pro-healing phenotype. In turn, Schwann cells migrated to the scaffold and remyelinated regenerating axons.

Recently, 3D printing has emerged as an advanced technique to fabricate biomimetic nerve scaffolds. This approach enables accurate control over the microarchitecture of the scaffold and, at the same time, optimizes the geometry of the implant to match a particular lesion site in a patient-specific fashion. A few attempts were recently reported. Joung et al. bioprinted an alginate microchannel scaffold with direct deposition of neural progenitor cells within each channel [100] (Figure 5E). The printed cells maintained viability for 14 days, and progenitor-derived neurons were seen to be active in calcium imaging. However, the scaffold was not tested in vivo, and it remains unclear whether it would have withstood implantation and integration into the host spinal cord.

In a later study, Koffler et al. used photopolymerization-based 3D printing to assemble highly accurate biomimetic scaffolds for the spinal cord composed from multiple microchannels [91] (Figure 5F,G). The scaffolds were tested in vivo in complete SCI lesions and were able to guide axons to grow in linear trajectories. When the scaffolds were loaded with NSCs and tested in prolonged in vivo experiments for six months, they were able to support functional recovery in a complete spinal cord lesion. Recently, we utilized 3D printing to fabricate soft biodegradable polyester scaffolds for spinal cord repair [62] (Figure 5B). Highly accurate sacrificial constructs were printed and a mixture of PLLA and PLGA was cast, and freeze-dried inside the construct. The fabrication process resulted in highly porous scaffolds (>95%) and over 50% open channel volume, showing significant improvements compared with previous microchannel scaffolds.

Another approach to reproducing nerve tissue microarchitecture is to use decellularized implants. This has been well studied for peripheral nerve injuries, including in clinical settings. Nerves can be harvested from deceased donors and decellularized to maintain only the ECM components of the nerve, making the implants non-immunogenic [72]. Upon implantation, the collagenous membranes of the decellularized graft serve as topographic cues for axons to follow during regeneration. However, decellularized grafts currently remain inferior to the gold standard autografts and are indicated in human patients only for small sensory nerves and with gaps of less than 3 cm [11].

However, decellularization techniques are continuing to improve. Recently a new method to decellularize allografts was reported, relying on elastase to reduce MHC expression [115]. These grafts were shown to be comparable to an autograft at a 3 cm gap. Additionally, storing the graft at 4 °C provided better results than −80 °C, likely due to protein denaturation in the freeze-thaw cycle. This discovery may have significant impact in the clinical setting, when attempting to replace autografts with off-the-shelf alternatives. Another recent study demonstrated that decellularized nerve matrix hydrogel can be reshaped in the topography of nerve tissue using freeze drying [116]. This technique allows more control on the implant measurements and the implant can be effectively incorporated with growth factors.

Despite the extensive use of engineered topographies for axonal alignment in multiple studies, the mechanism by which axons sense the scaffold topography and follow its path is not entirely clear. For example, in the case of microchannels, their size is typically significantly larger than the axon diameter. Yet, axons still manage to adopt linear growth patterns when regenerating into microchannel scaffolds. It is likely that several different mechanisms are involved in the sensing of different engineered topographies. The alignment of axons in accordance with scaffold topography can be explained to some degree by molecular signaling. Integrin receptors at the growth cone filopodia facilitate attachment to permissive substrates. These filopodia consist of bundles of filamentous F actin. The F actin filaments align with external anisotropic cues to minimize cell cytoskeletal distortion [117]. The F actin alignment then affects the directionality of axonal growth via regulatory and structural interactions present between the growth cone’s F actin and axonal microtubules. Nonetheless, these events, which take place at the level of the growth cone, likely provide only a partial explanation for how axons adopt the directionality of extracellular topographies, and continued research on this topic is warranted.

## 9. Drug Release

While biomaterial implants for nerve injuries are mainly designed to mimic the pre-injury ECM, they can also be manipulated to deliver novel therapies directly to the lesion site. This is particularly relevant in SCI, where implantation of a permissive substrate alone does not generate a significant functional recovery [62,91]. As an example, stem cells and other cell populations can be loaded into the biomaterial scaffold prior to implantation. Grafted cells can than secrete growth factors needed for regeneration [98]. Some cell populations can also produce and secrete ECM molecules, and some stem cell populations can differentiate into new neurons that integrate in the spinal cord circuitry and relay signals across the lesion [46,65]. Cell therapy for nerve and spinal cord injuries has been thoroughly reviewed in several articles [21,118,119,120,121,122,123,124]. An additional approach to enhancing the therapeutic potential of engineered scaffolds is to release growth factors or other drugs directly from the biomaterial and thereby avoid the potential risks of cell therapy.

Growth factors are the main therapeutic proteins delivered from biomaterial scaffolds. Several different growth factors have been delivered in nerve injuries, including BDNF, NGF, glial-derived neurotrophic factor (GDNF) and others [101,125]. As in the case of permissive ECM proteins, growth factors can be used to functionalize biomaterial surfaces in several techniques, including physical adsorption and chemical modification. Their supplementation for scaffolds implanted in the spinal cord have been shown to significantly increase regeneration and functional outcome. For example, Han et al. fused a collagen-binding domain to BDNF to enhance binding of the growth factor to collagen scaffolds [126]. This resulted in the sustained release of BDNF and improved locomotion and axonal regeneration upon implantation to a partial SCI model in rats. The supplementation of growth factors also can be used beyond their direct effect on host spinal cord tissue. A series of studies used a cocktail of 4–10 growth factors mixed in fibrin hydrogel designed to support neural stem cell engraftment in partial and complete SCI [46,65,66,127]. The growth factor cocktail had a dramatic effect on the survival of NSCs and consequently on the number of axons extending from stem cell- derived neurons.

Despite the significant effects of growth factors on axonal regeneration, not all neurons respond similarly to a specific type of growth factor. For example, NGF and GDNF have differential expression profiles in motor and sensory nerves [101]. This principle was applied in a study by Johnson et al. to generate and reconstruct specific axonal pathways within an injured sciatic nerve in rats [101]. The nerve injury in the study extended into a bifurcation of the sciatic nerve to a sensory branch and a motor branch. GelMA was 3D printed to form an anatomical scaffold with the geometry of lost nerve tissue (Figure 5H). NGF and GDNF were selectively printed in the appropriate nerve pathway (Figure 5I,J). An NGF gradient was placed to selectively regenerate the sensory branch, and the GDNF gradient was located to regenerate the motor pathway. The supplementation of growth factors in this study resulted in improved functional recovery. However, the selectivity of each gradient to sensory or motor axons was not shown. Nonetheless, this study is an important demonstration of the potential of 3D printing to specifically localize therapeutic proteins in biomaterial implants.

Alongside growth factors, other proteins and pharmacological agents have been loaded into biomaterial scaffolds. For example, chondroitinase ABC has been incorporated into biomaterial scaffolds in several studies to degrade components of the inhibitory glial scar and increase neural regeneration [128,129]. Small molecules have also been released for biomaterial matrices to assist in nerve repair. Recently four small molecules, LDN193189, SB431542, CHIR99021, and P7C3-A20 were incorporated into an injected collagen hydrogel tested in a complete SCI model [56]. The small molecules were able to recruit NSCs to the lesion site, which differentiated into new neurons. Their supplementation also resulted in better functional recovery and larger amplitude in neurophysiological assessments. While biomaterial implants are often compared to nerve autografts and are designed to match their level of performance, they can be tailored to deliver unique therapeutic agents which are not present in a standard autograft. Accordingly, future studies may generate biomaterial implants that surpass the efficacy of nerve autografts and enable the treatment of severe nerve injuries that are currently incurable.

## 10. Clinical Translation

From a clinical perspective, it is difficult to study SCI and PNI together. Using biomaterials to repair the spinal cord seems like a distant goal for which only a handful of human trials have been conducted. The multiple extrinsic and intrinsic regeneration inhibitors in the injured spinal cord makes it much more difficult to repair compared with peripheral nerves, and biomaterials on their own may not offer a feasible solution in the near future. By contrast, in the case of PNI, where the capability of axons to regenerate is significantly higher, some biomaterial solutions are already in clinical use.

While the gold standard repair for PNI is an autograft, some biomaterial implants are in routine clinical use and are indicated for nerve injuries with small gaps (Table 1). For example, collagen and polyester hollow tube conduits have been tested in clinical trials and are available as commercial products. They are usually used for <3 cm gap injuries in digital nerves. Bushnell et al. used a collagen hollow tube to repair digital nerve injuries of 12 patients [130]. A two-point discrimination test was good or excellent in eight of nine patients who continued follow-up. Recently, a clinical trial examined a collagen hollow conduit filled with collagen fibers to mimic the nerve ECM structure [131]. The findings showed that when applied to sensory nerve injuries at the level of the wrist or below it, the use of the collagen implant was equivalent to an autograft. Weber et al. evaluated 98 subjects with 136 nerve transactions in the hand. Forty-six nerves were repaired with a polyglycolic acid (PGA) implant, and fifty-six nerves received a standard repair that included either an end-to-end repair or an autograft [132]. The findings showed no differences between the groups. Surprisingly, in a subgroup of injuries with gaps of over 8 mm that received either a PGA conduit or an autograft, better results were observed in the PGA group.

Decellularized nerve implants from cadaver donors have also been evaluated in clinical trials and are available as commercial products. They may be somewhat more appealing to use as they possess the collagenous inner membranes of the nerve and potentially could be more efficient in guiding axons in accordance with pre-injury trajectories. He et al. compared decellularized nerve implants to standard nerve repair in digital nerves [133]. In this study, 72 patients received a decellularized implant and 81 patients received standard repair. The findings showed that when the graft length was <5 cm, the decellularized implant was non- inferior to an autograft. It is not entirely clear whether decellularized implants or hollow tubes are superior. However, a study in rats showed that decellularized implants outperformed collagen conduits for both 14 mm and 28 mm gap injuries [72]. Still, with advances in production and optimization of biomaterial implants, this may change in the future.

Following the progress of PNI biomaterial implants and the encouraging results in small gap injuries, several commercial products are now available and have been recently evaluated in a number of studies [134,135,136]. For example, Neuragen and Neuroflex are two commercially available collagen implants approved by the food and drug administration (FDA) for PNI repair [142]. Haug et al. evaluated the Neuragen implant for repair of digital nerve defects of up to 26 mm. Good functional outcome was observed in the majority of patients [135]. Neurolac and Neurotube are examples of FDA approved polyester implants [142]. Neurotube was also evaluated for the rapier of the facial nerve and was found to be efficient in gaps of less than 3 cm [137]. Axogen Avance is an FDA approved decellularized graft. It was recently evaluated by Rbia et al. in 18 patients and compared with the Neuragen implant. No clear differences were found between these two implants with good outcome observed in gaps <25 mm.

Despite being useful for small-gap sensory nerve injuries in human patients, biomaterial implants are still limited in their utility for many types of PNI. Their efficiency in large-gap injuries has not been established [11]. Future studies and progress in the field should focus on enhancing the effectiveness of biomaterials to serve as autograft replacements for large-gap injuries. Currently, synthetic scaffolds for PNI are in clinical use only as hollow tube implants. Microchannel scaffolds that mimic the collagenous membranes of the nerve and can be loaded with cellular and molecular therapies may provide a superior alternative to hollow tubes in large-gap injuries. However, microchannel scaffolds may still need to undergo improvements before being translated to clinical use. Particularly, the open volume of these implants should be increased. Novel fabrication techniques, including more accurate 3D printing, may assist in future studies to reduce the scaffold wall thickness and increase open volume for axonal regeneration.

Attempts to use biomaterials in clinical settings for SCI are less common and have achieved more limited results (Table 1). Theodore et al. reported the first in human implantation of a bioresorbable porous scaffold for acute and functionally complete SCI [139]. The report was a single-patient case study where, at three months post-implantation, substantial neurological improvements were observed. However, it could not be confirmed whether these improvements were solely a result of the implanted scaffold or also partially related to surgical decompression. A different study examined a collagen fiber scaffold with bone marrow cells implanted in five patients with chronic and functionally complete SCI [140]. Patients underwent a surgical resection of fibrotic tissue in the lesion site and transplantation of the implant. Follow-up was continued for 12 months. Patients showed autonomic improvements and electrophysiological improvements; however, other meaningful motor or sensory improvements were not reported. In a later study by the authors, the same scaffold with umbilical cord mesenchymal stem cells was implanted in eight patients. Some modest positive results were observed in motor function and level of sensation [141].

While some improvements have been observed in the implantation of biomaterials in clinical trials of SCI, to the best of our knowledge there are currently no published, large-scale clinical studies with significant positive outcomes to support widespread biomaterial treatment for routine clinical use. The results in recent preclinical studies are encouraging and may suggest that clinical translation should be attempted [75,76,143]. However, the progression to clinical trials with biomaterials should always be carried carefully. Examples for premature human studies with biomaterials have been reported [144]. Specifically, the safety of spinal cord biomaterial implants must be properly evaluated and monitored before and during clinical trials. The safety of incorporating stem cells into biomaterials intended for spinal cord repair should also be thoroughly confirmed in preclinical studies and an appropriate target population should be carefully chosen for early human studies [145]. In our view, biomaterials may become useful for SCI as part of combinatorial treatments where several interventions are delivered together, including growth factors, cellular treatments, electrical stimulation, and biomaterials. Focusing on the optimization of individualized biomaterials for delivering cellular and pharmacological interventions may hold promise for productive clinical translation.

## Figures and Tables

**Table 1:** Human studies of biomaterial implants in PNI and SCI.

Type of Injury	Gap Size	Biomaterial Implant	Number of Patients Treated	Outcome	Reference
Digital nerve injury	10–20 mm	Collagen	12	Sensory improvements	[130]
Sensory injury below the wrist	≤30 mm	Collagen	49	Equivalent to autologous graft	[131]
Sensory injury below the wrist	≤30 mm	PGA	46	Equivalent to standard repair or superior in some subgroups	[132]
Digital nerve injury	10–50 mm	Human acellular nerve graft	72	Non-inferior to autologous grafts	[133]
Digital nerve injury	<25 mm	Collagen/Acellular graft	19 (Collagen) 18 (Acellular)	Similar outcome between collagen and acellular graft	[134]
Digital nerve injury	≤26 mm	Collagen	35	Good functional outcome in majority of cases	[135]
Digital nerve injury	1–50 mm	PGA with collagen scaffolding	20	Meaningful recovery in 90% or repairs	[136]
Facial nerve injury	10–30 mm	PGA	7	Some muscle recovery in the majoroty of patients	[137]
Median nerve injury in distal forearm	30 mm	Chitosan-PGA	1	Significant motor, sensory and electrophysiological improvements	[138]
T11 Spinal cord injury	10 mm	PLGA conjugated to Poly L lysine	1	Partial motor, sensory and autonomous recovery	[139]
C6-T12 Spinal cord injury	5–45 mm	Collagen	5	Partial autonomous recovery	[140]
C6-T10 spinal cord injury	13–50 mm	Collagen	8	Mild motor, sensory and autonomic recovery	[141]
